# Hybrid Task Coordination Using Multi-Hop Communication in Volunteer Computing-Based VANETs

**DOI:** 10.3390/s21082718

**Published:** 2021-04-12

**Authors:** Abdul Waheed, Munam Ali Shah, Abid Khan, Carsten Maple, Ikram Ullah

**Affiliations:** 1 Department of Computer Science, COMSATS University Islamabad, Park Road Tarlai Kalan, Islamabad 45550, Pakistan; mshah@comsats.edu.pk (M.A.S.); ikram.comsats.cs@gmail.com (I.U.); 2 Department of Computer Science, Aberystwyth University, Ceredigion SY23 3DB, UK; abk15@aber.ac.uk; 3 Secure Cyber Systems Research Group, WMG, University of Warwick, Coventry CV4 7AL, UK; CM@warwick.ac.uk

**Keywords:** volunteer computing, vehicular networks, boundary relay nodes, resource utilization, task offloading, multi-hop communication, latency

## Abstract

Computation offloading is a process that provides computing services to vehicles with computation sensitive jobs. Volunteer Computing-Based Vehicular Ad-hoc Networking (VCBV) is envisioned as a promising solution to perform task executions in vehicular networks using an emerging concept known as vehicle-as-a-resource (VaaR). In VCBV systems, offloading is the primary technique used for the execution of delay-sensitive applications which rely on surplus resource utilization. To leverage the surplus resources arising in periods of traffic congestion, we propose a hybrid VCBV task coordination model which performs the resource utilization for task execution in a multi-hop fashion. We propose an algorithm for the determination of boundary relay vehicles to minimize the requirement of placement for multiple road-side units (RSUs). We propose algorithms for primary and secondary task coordination using hybrid VCBV. Extensive simulations show that the hybrid technique for task coordination can increase the system utility, while the latency constraints are addressed.

## 1. Introduction

With the rapid advancements in technologies and ongoing urbanization, the number of vehicles and applications is growing rapidly. According to a recent Green Car report [1], the number of vehicles on the road were 1.2 billion in 2014 and is set to reach 2 billion by 2035. This huge number of vehicles results in a tremendous increase in traffic, especially during peak hours, which is an extensive global phenomenon. In the United States, people travelled 6.9 billion extra hours due to traffic congestion in 2014 [2]. During such rush hours, vehicles stuck in congestion can access remote servers to fulfill the requirements of task execution. Using wireless communication, these vehicles are able to act as nodes in autonomous self-organized networks, known as vehicular ad-hoc networks (VANETs). In these networks, vehicles can connect using a dedicated short-range communication (DSRC) service, for communication between vehicle-to-vehicle (V2V) and vehicle-to-road-side unit (RSU) (V2R) [3].

Mobile Cloud Computing (MCC) is a promising paradigm that provides vehicles with an opportunity to offload the computational or storage tasks to remote cloud servers. It provides ubiquitous access to incorporated resources offered by a variety of cloud computational and storage technologies. Users gain the opportunity of executing computationally-intensive tasks whose performance would be hindered by the computational capability of a single user [4]. Vehicular Cloud Computing (VCC) is a similar paradigm that additionally uses the computational capabilities of vehicles in the form of vehicular clouds (VCs). Usually, accessing remote clouds has some disadvantages such as high latency and infrastructure costs. These high latencies are not convenient for delay-sensitive applications. Offloading to remote clouds is not practicable for services and applications that solely depend on time and place. To address these place-bound services the best position for computation is proximal to users [5].

Edge computing is an architecture that brings computation and storage capabilities at the edge of the network—in user proximity. It reduces the latency incurred due to distant clouds, and can fulfil the requirement in delay-sensitive applications. It also reduces the size of data moved through the network [6]. Mobile Edge Computing (MEC) has brought an opportunity to deploy servers with significant computational resources at the edge, in the proximity of users. With the emergence of 5G radio access networks, MEC provides a promising solution of lowering latency for task offloading. It was also benefits in task offloading to MEC servers from vehicles that are equipped with wireless and cellular connectivity [7]. Vehicular Edge Computing (VEC) similarly brings computation to the edge of the network, enabling multiple vehicles to offload their tasks to servers at RSUs. Contrary to MEC, the distinctive features of VEC are the dynamic topology changes in vehicular networks due to the speed of vehicles. In VEC, RSUs act as VEC servers which are responsible for collecting, storing, and processing data where vehicles have different communication, computation, and storage resources. Due to the constrained resources or critical nature of the applications, vehicles offload computation-intensive and delay-sensitive tasks to the VEC servers, which can substantially lower the latency and efficiently relieve the burden on backhaul networks [8].

Like edge computing, fog computing also provides services at devices near end users. Fog computation avoids unnecessary network jumps and provides improvements in latency for delay-sensitive applications [9]. Vehicular Fog Computing (VFC) is an emerging paradigm that came into existence with the integration of fog computing and vehicular networks [10]. There are no separate dedicated servers but dynamic clusters of vehicles to decrease the latency while taking advantage of abundant computational resources. It relies on the strategy of collaboration with nearby vehicles, instead of depending on the remote dedicated servers. This strategy shortens deployment costs and delays. According to [11], the three layers of VFC architecture are the abstraction, policy management and application services layers. VFC provides cooperation between cloud computing and fog computing in vehicular networks, to realize benefits for both user vehicles and intelligent transportation systems (ITS). Additionally, the user experience can be improved without any surplus load on V2V communication through the use of smart fog nodes at significant data sensing points [12]. When processing is pushed from the edge of the network to the user layer involving actuators and sensors, it further decreases the latency and increases the self-reliance of the system [13].

The use of processing capabilities within user devices at the user layer has been termed as mist computing [14]. This represents the first computing locations in the user networks. It has also been labelled as Things Computing since it extends the computing and storage processing to the things. Volunteer computing is an approach to distributed computing where users volunteer their idle computing resources to help in solving computation-intensive scientific problems. The basic motive for volunteer computing was to find a free-of-cost model for solving computation-intensive problems. It also solves the problem of the wastage of surplus resources in any computing device. Therefore, volunteer computing is seen as the premium option for utilizing resources in any connected computing devices. When vehicles are stuck in congestion for a long time, accessing remote servers from various vehicles induces a great load on the Internet and remote servers for task offloading. Volunteer Computing-Based Vehicular Ad-hoc Networking (VANET), abbreviated as VCBV, is a new approach that is used for task execution and resource utilization in VANETs [15].

In this article, we propose a hybrid task execution method in VCBV that exploits the infrastructure and ad-hoc coordination simultaneously for task execution and resource utilization. Hybrid task execution utilizes the resources of vehicles in a multi-hop fashion which increases the resource utilization by adding more resources including those lying out-of-range for the job coordinator. We consider a congestion scenario where most of the resources are underutilized and task offloading to third-party service providers is at peak, due to leisure timings for drivers and passengers. In this scenario, where tasks are initiated from an RSU and coordinated with volunteer vehicles and extended in an ad-hoc fashion, we formulate the problem and design the algorithm to solve the computation offloading and resource utilization issues. The main contributions of our work are summarized as follows.

(1) We propose a hybrid task coordination model for job execution and surplus resource utilization. This model consists of the infrastructure and ad-hoc task coordination simultaneously.
(2) We propose a method to identify the boundary relay vehicles to enhance the region of resource utilization without using additional RSUs.
(3) We design and validate the primary and secondary task coordination algorithms.

The rest of this article is structured as follows: In Section 2, we discuss the background of task offloading in vehicles and related paradigms. Section 3 introduces hybrid VCBV coordination. In Section 4, we describe the system model along with the communication and computation models. Problem formulation regarding cost avoidance is presented in Section 5, and the proposed models and algorithms are explained in Section 6. The performance analysis is presented in Section 7 before the article is concluded in Section 8.

## 2. Related Works

With significant advances in technologies, new applications such as augmented/virtual reality and autonomous driving have developed. These applications have high computational requirements for execution. Unfortunately, computational and storage resources in a single vehicle are not capable of performing these executions in a timely manner. The task offloading concept has been introduced to address these limitations in vehicles. In this concept, computation-intensive tasks are fully or partly migrated from vehicles to resource-rich remote servers/vehicles. In this section, the offloading of a task is reviewed. We describe task offloading hosts in two categories i.e., dedicated servers and cluster of vehicles with surplus resources. The first category where tasks are offloaded to remote servers includes MCC, MEC, and VEC whereas the second category includes VCC and VFC.

MCC, the integration of cloud computing with mobile computing devices, provides computing and storage services taking full advantage of cloud computing. The basic functionality of computation offloading is the decision about the task to be offloaded or not, and the server where it would be offloaded [16]. Connectivity and availability of clouds are two requirements for effective task offloading, while the level of resources of bandwidth and network access latency affect the decision of task offloading. Offloading computational tasks at distant clouds may bring additional communication overhead affecting the quality of service (QoS). Algorithms have been developed that use a game-theoretic approach, to enable the user to decide about the offloading decision to the device itself, cloudlet, or remote cloud [17]. Wu et al., [18] proposed an energy-efficient algorithm based on Lyapunov optimization which optimizes energy efficiency by switching the offloading between local, cloud, and cloudlet computing. Guo et al. [19] presented an efficient strategy for dynamic offloading and resource scheduling for optimization of consumed energy and latency. This problem was formulated to minimize energy consumption and application completion time. A real testbed is used for experimentation and validation which shows the efficiency of the proposed scheme over the existing schemes. However, offloading to the remote cloud and increased load can affect the performance so as to make the strategy unsuitable for delay-sensitive applications. Attempting more than one optimization objective was also considered for the efficiency in computational offloading. A multi-site offloading solution was proposed which addresses two average execution time and energy. For this multi-objective task offloading scheme, an algorithm was designed to address energy and execution time with bandwidth condition consideration [20].

To address the higher latency incurred due to distant clouds, MEC involves the proximal placement of servers. The key idea behind MEC functionality is to provide services at base stations where computation-intensive and energy-consuming tasks are offloaded for execution. Usually, cellular communication services, such as 4G or 5G, are used to connect to the MEC server. Both partial and full offloading options for migration are utilized. When some parts of the application are offloaded to the server, it is partial offloading whereas, in full offloading, all the parts of an application are offloaded to the MEC server. Since MEC uses proximal servers to minimize the delay that occurred due to distance cloud, it is also suitable for computation offloading in vehicular networks [21]. The use of MEC in vehicular networks can improve interactive responses during the computational offloading for delay-sensitive applications. However, the additional offloading load from dense traffic vehicles other than the mobile devices for MEC servers may lead to optimum makespan [22,23]. In VEC [24,25], the computational and processing tasks are also offloaded from the vehicles to proximal servers. In earlier research, reputation management [26] and low latency in caching [27] have been discussed. In this work a multi-objective VEC task scheduling algorithm is proposed for task offloading from user vehicles to MEC vehicles. Extensive simulations show reduced task execution time for task offloading with high reliability [28]. A mobility-aware task offloading scheme [29] and collaborative computation offloading and resource allocation optimization scheme [30] are proposed for computation offloading in MEC.

Dai et al., [31] considered the tasks of offloading and load balancing jointly. JSCO, a low complexity algorithm was proposed and used to address the problem of server selection and task offloading. Numerical analysis demonstrated the effectiveness of the proposed solution. The main problem area explored was the reduced link duration of users with static servers. The load on communication and computation resources can be effectively managed through the use of scheduling algorithms in distributed environments.

Fog computing and vehicular network approaches can be combined to utilize surplus resources in vehicles through the use of vehicular fog nodes. In VFC, computational task offloading can be performed using moving or parked vehicular fog nodes. Hou et al. [32] presented the concept of VFC where vehicles are utilized as infrastructure. Their approach is based on the collaborative utilization of communication and computation resources of several edge devices or end-user devices. Due to the wide geographical distribution of fog computing, VFC is a better option for delay-sensitive applications in vehicular networks [33]. In [34], VFC is shown as comprising three layers, namely cloud, cloudlet, and fog layers, which cooperate for the network load balancing.

Resource allocation in VFC is a major challenge since the resources are geographically distributed. Therefore, it is necessary to allocate the resources appropriately to minimize the service latency. For applications having diverse QoS requirements, the admission control problem is solved using a theoretical game approach. With the help of the proposed scheduling algorithm, QoS requirements and scalability are achieved [35]. In another work [36], public service vehicles are used as fog nodes for task offloading using a semi-Markov decision process. To increase the long-term reward and gain the optimal allocation of resources, an application-aware policy is used for offloading. Zhou et al., [37] presented a model to minimize the load on the base station by using the underutilized resources of vehicles with the help of an efficient incentive mechanism and by using the stable matching algorithm based on pricing.

In an effort to efficiently park vehicles, vehicular fog computing has been employed [38]. In this work, the scheme is introduced to guide the vehicles for parking places with fog nodes and smart vehicles. Efficiency is achieved with the help of parked and moving vehicles with surplus resources. The participating vehicles in service offloading were incentivized with monetary rewards. When task offloading, total delay comprising communication and computation delays can be critical for delay-sensitive jobs. For VFC systems that provide offloading services, the long-term reward is very important; this depends on resource availability, heterogeneity, transmission, and computation delays. Wu et al. formulated a model named SMDP which consists of the components required for task offloading [39]. With the help of an iterative algorithm based on the 802.11p standard, the target of maximal reward was achieved. 

Vehicles with automated driving capabilities must have accuracy and sensing coverage. To overcome the limitations of computing resources in a single vehicle, Du et al. [40] proposed Li-GRU. The simulations show the improvement in sensing and coverage of a single vehicle. Parallel computing is an effective process for the on-time completion of tasks. Resource aware based parallel offloading [41] was proposed to find suitable nodes for task offloading. The effectiveness of the proposed scheme is validated through simulations.

In this article, the idle resources of vehicles stuck in traffic are utilized using hybrid VCBV to execute jobs offloaded to a central entity (RSU) from vehicles, pedestrians, or an internet of things (IoT) device. The objective of this article is to fully utilize the resources in a multi-hop fashion without using additional infrastructures as well as avoid monetary costs payable to third party vendors.

## 3. Hybrid Volunteer Computing Based VANET

Volunteer computing is a type of distributed computing in which any computing device can share its surplus computing resources voluntarily to perform computation-intensive tasks. Using volunteer computing, resource-intensive tasks can be performed without the use of expensive computing infrastructure. VC has previously been applied successfully in a variety of domains to solve computation-intensive tasks [42]. The number of vehicles on roads is growing rapidly and the resources of vehicles in the form of on-board units (OBUs)—the small computers mounted on vehicles for communications and computation—are often left idling, and can be utilized with the help of volunteer computing. To utilize the surplus resources in VANETs, volunteer computing and VANET are merged into a new architecture named VCBV [15]. The computing power of vehicles can be utilized without requiring connectivity to the Internet, whether vehicles are parked or idling in congestion. Amjid et al. [43] used volunteer computing over VANETs to support autonomous vehicles, utilizing resources through a centralized job manager. A number of algorithms, differentiated by node-registration, were evaluated for job completion rate, latency and throughput, using NS2 and SUMO. However, hybrid coordination using infrastructure and ad-hoc networking simultaneously for resource utilization has not yet been considered. Further, the impact of using volunteer computing in VANETs in terms of makespan and monetary cost for a job has not been evaluated.

**Figure 1:**
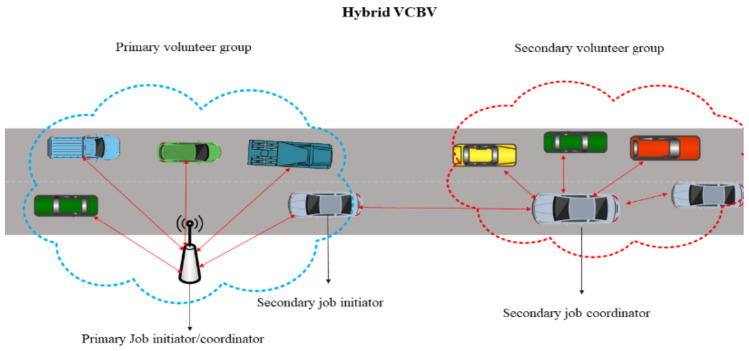
Hybrid VCBV.

In this article, we use hybrid VCBV to utilize the resources of vehicles in congestion. The major advantage of this type of computing is to utilize the resources within VANETs, thereby reducing latency. In hybrid VCBV, the RSU maintains a queue of jobs received from pedestrians, vehicle drivers, passengers, or even from IoT devices. DSRC communication is used for initial offloading to the RSU. The RSU arranges the jobs and decides for selection of jobs for coordination. The RSU receives notification of willingness from volunteers located in its communication range and partitions the selected jobs into the appropriate number of tasks. In the hybrid VCBV scenario, an RSU can select another job coordinator, which can be another RSU or a willing volunteer vehicle. This second coordinator is known as the secondary coordinator and can be found using boundary relay vehicles. The primary difference between hybrid and other types of VCBV is that hybrid uses both RSU and ad-hoc task coordination simultaneously as shown in Figure 1.

## 4. Hybrid VCBV System Model

In this section, we present our proposed hybrid VCBV architecture and elaborate on the system model in detail. The important notations used in this paper are presented in Table 1.

### 4.1. Network Model

**Figure 2:**
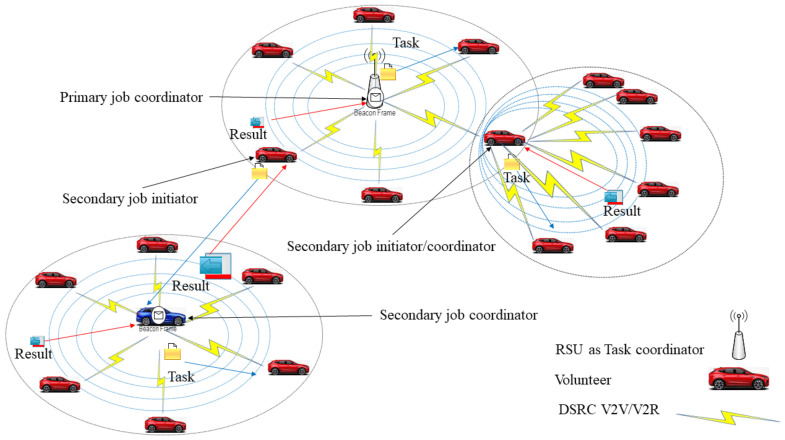
System Model for Hybrid VCBV.

The scenario considered in this paper is of vehicles in congestion that voluntarily process tasks. The network model of hybrid VCBV can be explained in Figure 2. In the scenario, there is a primary job initiator/coordinator, a secondary initiator/coordinator and volunteers. The details are as follows:

#### 4.1.1. Primary Job Initiator

In hybrid VCBV, a vehicle, RSU, pedestrian, or IoT device having some job to be performed acts as a job initiator. The job initiator sends a job (or jobs) to the RSU for further coordination. The job initiator and task coordinator might be the same or different depending upon the situation. 

#### 4.1.2. Primary Task Coordinator

In hybrid VCBV, an RSU is usually the primary task coordinator, receiving jobs from the primary job initiator. It then schedules the jobs according to priority/incentives, and obtains willingness notifications from volunteers. After receiving the willingness, it partitions the job into the required number of tasks and coordinates the tasks between suitable volunteers.

#### 4.1.3. Volunteer Vehicles

Volunteer vehicles are the vehicles present in the communication range of a task coordinator that are willing to participate in volunteer computing. In the aforementioned scenario, these vehicles are in congestion and can be used as volunteer resources to perform computational tasks. A job is partitioned into some tasks according to available volunteer resources. We assume there are n vehicles in the communication range of job initiator (RSU/vehicle) willing to serve as volunteers. We denote a set of vehicles as $V=\left\{1,2,3,\ldots n\right\}.$

#### 4.1.4. Secondary Job Initiator

**Figure 3:**
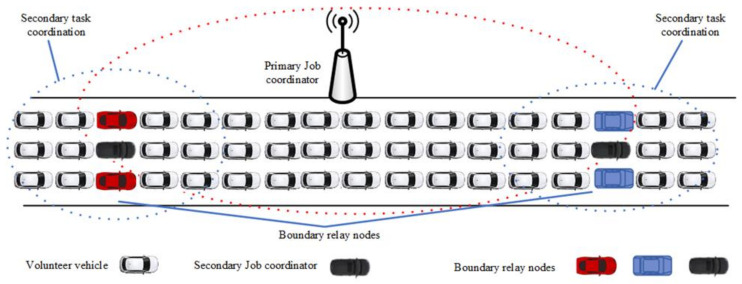
Boundary relay nodes.

Boundary relay nodes from the n volunteers can play the role of secondary job initiators to maximize resource utilization and minimize the makespan incurred during job execution. If the distance of vehicle i from the primary job coordinator is larger than the distance between the coordinator and all other volunteer vehicles, then vehicle is termed to be a boundary relay node as shown in Figure 3. Let $R_{r}$ be the communication range of RSU and $D_{\mathrm{ir}}$ be the distance of vehicle i and RSU. Node i would the boundary relay node if $\delta_{ir}$ is minimum positive value for all i∈V:

(1) $$\delta_{ir}=R_{r}-D_{ir}$$

from all boundary nodes, i and j two boundary nodes would be selected as secondary job coordinators which have the maximum distance $D_{ij}$ between them.

#### 4.1.5. Secondary Task Coordinator

Either the secondary job initiator obtains the willingness of volunteers in its communication range and acts as coordinator, or it forwards the task to another vehicle or an RSU which can then acts as a task coordinator. This type of coordinator is termed a secondary task coordinator and accumulates further volunteers, resulting in an increase in resource utilization and optimized makespan.

### 4.2. Communication Model

In the scenario we have presented, it is assumed that vehicles are stopped and use the IEEE 802.11p standard for communication between V2V and V2R, providing 3 Mbps to 27 Mbps data rates over 10 MHz bandwidth [44]. Request-to-send (RTS) and clear-to-send (CTS) are both mechanisms used to reduce collisions in task transmission and result gathering. The data transmission rates between V2V and V2R using Shannon’s formula are as follows:

(2) $$R_{t}^{V2V}=b_{V2V}^{\mathrm{cm}}\log_{2}\left(1+SNR_{V2V}\right)$$

(3) $$R_{t}^{V2R}=b_{V2R}^{\mathrm{cm}}\log_{2}\left(1+SNR_{V2R}\right)$$

where $R_{t}$ is the data transmission rates for the wireless channel, b is bandwidth allocated and SNR is signal-to-noise ratio respectively. SNR can be found using the following formula:

(4) $$SNR=\frac{Pd^{-\alpha }}{I_{V2V}+\sigma^{2}}$$

where P is the received signal power of the channel, I is interference, and σ is the noise power. α is the path loss component that depends on distance d between two communicating entities which can be found using the following formula:

(5) $$d_{ij}=\sqrt{{\left(x_{i}-x_{j}\right)}^{2}+{\left(y_{i}-y_{j}\right)}^{2}}$$

The data transmission latency between RSU and a volunteer vehicle “i” is given by the following equation, where $tp_{i_{IS}}$ is task input size allocated to vehicle “i”.

(6) $$T_{Ri}^{t}=\frac{tp_{i_{IS}}}{R_{t}^{R2V}}$$

### 4.3. Task Model

Here we present a task model for hybrid VCBV. Each job can be partitioned into a number of distinct tasks of the same sizes which may be carried out on OBUs. Every task is presented in the form of a tuple $tp_{i}=\left\{tp_{i_{ID}},\;tp_{i_{IS}},\;tp_{i_{CR}}\right\}$, where “i” represents the vehicle ID from set “V” willing to participate in task execution, $tp_{i_{ID}}\;$is separate identity allotted to each partitioned task, $tp_{i_{IS}}$ describes the input size (in bits) of the task sent and $tp_{i_{CR}}$ shows the computational resources required (CPU cycles per bit) to complete the task $tp_{i}$. Task processing mainly relies on its input size ($tp_{i_{IS}}$) and computational requirement ($tp_{i_{CR}}$) which is also known as the complexity factor. This factor is crucial to explain the distinct computational requirements. Some tasks, such as applying filters on an image, normally require fewer CPU cycles than applying an algorithm for face detection in a video [45].

### 4.4. Vehicle Computation Model

The makespan incurred for a job consists of three types of delay for a single task, namely transmission time, computation time, and results collection time. Transmission time depends upon the transmission rate of the channel and the size of the task. The computation time of the task relies on two elements which are the computational requirements of a task and the computational capability of the volunteer vehicle. The third type of delay is the result collection time from the volunteer to the RSU which is dependent on the size of the output data. The time taken for a task to complete its execution on a volunteer vehicle is shown in the following equation:

(7) $$T_{{\mathrm{tp}}_{i}}^{\mathrm{comp}}=\frac{tp_{i_{IS}}\times tp_{i_{CR}}}{C_{i}^{Veh}}$$

The total time to transmit and execute a task on a volunteer vehicle is shown in the following equation:

(8) $$T_{tp}{}_{i}=\frac{tp_{i_{IS}}}{R_{t}^{R2V}}+\frac{tp_{i_{IS}}\times tp_{i_{CR}}}{C_{i}^{Veh}}$$

The total makespan for a job j to complete with the help of n vehicles is as follows:

(9) $$T_{j}^{Veh}=\overset{n}{\underset{i=0}{\sum }}\frac{tp_{i_{IS}}}{R_{t}^{R2V}}+\underset{i∊n}{\max}\frac{tp_{i_{IS}}\times tp_{i_{CR}}}{C_{i}^{Veh}}+\overset{n}{\underset{i=0}{\sum }}\frac{D_{o_{i}}}{R_{t}^{R2V}}$$

Similarly, the average execution time for all m jobs is as follows:

(10) $$T_{avg}^{Veh}=\frac{\sum_{j=0}^{m}T_{j}^{Veh}}{m}$$

### 4.5. Cloud Computation Model

The offloading from vehicle to cloud includes transmissions from the vehicle to the RSU and then from the RSU to the cloud. Vehicles use DSRC for connectivity to the RSU and backhaul links such as fiber and core networks are used to offload jobs from an RSU to cloud servers placed thousands of miles away [30]. Transmission time includes offloading input tasks and getting back the results. Total time to offload and execute a job *j* on cloud is $“T_{j}^{CC}”$ which is expressed as follows:

(11) $$T_{j}^{CC}=\frac{\sum_{i=1}^{n}tp_{i}{}_{IS}}{R_{t}^{R2V}}+\frac{\alpha \times \sum_{i=1}^{n}tp_{i}{}_{IS}}{R_{t}^{R2C}}+\frac{\beta \times \sum_{i=1}^{n}tp_{i}{}_{IS}\times tp_{i}{}_{CR}}{C^{CC}}+\frac{D_{o}}{R_{t}^{R2C}}+\frac{D_{o}}{R_{t}^{R2V}}$$

where α and β are constants and $D_{o}$ is the output data size. For the aforementioned scenario, it is assumed that all the jobs are already at the RSU.

Therefore:

(12) $$T_{j}^{CC}=\frac{\alpha \times \sum_{i=1}^{n}tp_{i}{}_{IS}}{R_{t}^{R2C}}+\frac{\beta \times \sum_{i=1}^{n}(tp_{i}{}_{IS}\times tp_{i}{}_{CR})}{C^{CC}}+\frac{D_{o}}{R_{t}^{R2C}}$$

Similarly:

(13) TavgCC=∑j=0mTjCCm

### 4.6. Edge Computation Model

Edge servers are placed at RSUs installed alongside the roads, and play the role of wireless access points, and are smaller but closer computation and data centers compared to cloud servers. After receiving a job from a vehicle, the RSU places the job in the queue and executes it in turn. In the aforementioned scenario, we assume that all jobs are present in the queue of an RSU. Therefore, the computation time for job *j* at the edge as follows:

(14) $$T_{j}^{Edge}=\frac{\gamma \times \sum_{i=1}^{n}\left(tp_{i}{}_{IS}\times tp_{i}{}_{CR}\right)}{C^{Edge}}$$

(15) TavgEdge=∑j=0mTjEdgem

### 4.7. System Utility Function

In this subsection, we define a logic function named the system utility function ($S_{uf}$) which depends upon latency and monetary cost, two important metrics for task offloading. Since low latency and costs are requirements of efficiency for task offloading, this system utility function increases monotonically with a decrease in latency or the cost paid. This function represents user satisfaction:

(16) $$S_{uf}^{j}=\frac{1}{\ln\left[T_{j}+\psi +\theta \times P_{c}\times \sum_{i=1}^{n}\left(tp_{i}{}_{IS}\times tp_{i}{}_{CR}\right)\right]}$$

where $P_{c}$ is price coefficient and θ and ψ are weight constants.

Similarly:

(17) Sufavg=∑j=0mSufjm

## 5. Avoiding Costs Paid to Third-Party Vendors

We formulate the optimization problem of lowering the makespan for task execution and considering the monetary cost at the same time. According to already explained communication and computation models, the system optimization problem relies on these two factors. Strategies lacking balanced resource allocation can affect the performance of the model which can raise the offloading latency while comparing to local computing. The optimization includes the minimization of makespan while comparing with a benchmark of total job execution time at a single vehicle. The optimization goals are to minimize job execution time, the cost paid to third party venders and restrict the makespan to benchmark. These optimization objectives are as follows:

(18) $$O_{1}:\;min\overset{m}{\underset{j=0}{\sum }}T_{j}$$

(19) $$O_{2}:max\overset{m}{\underset{j=0}{\sum }}S_{uf}^{j}$$

(20) $$O_{3}:\;\overset{n}{\underset{i=1}{\sum }}T_{i}<\frac{\sum_{i=1}^{n}tp_{i_{IS}}\times tp_{i_{CR}}}{C^{Veh}}$$

The solution to our problem is based on the achievement of these aforementioned objectives while identifying the possible constraints. If any coordination algorithm fulfils these objectives while handling the constraints, it will be considered as a suitable algorithm. Computation and communication constraints need to be satisfied by the proposed algorithm. The computations performed by vehicles cannot exceed the resource it owns. The link expiration time (LET) between the job coordinator and the volunteer vehicle must not be less than the time taken to complete the task execution by the volunteer. The task transmission time of offloading to volunteers or cloud must not exceed the computation time at the edge server. All these constraints are shown as follows:

(21) $$C1:\;tp_{i_{IS}}\times tp_{i_{CR}}<C_{i}$$

(22) $$C2:LET<\frac{tp_{i_{IS}}\times {tp}_{i_{CR}}}{C_{i}}$$

(23) $$C3:\sum_{i=1}^{n}\frac{tp_{i_{IS}}}{R_{t}^{R2V}}<\frac{\gamma \times \sum_{i=1}^{n}\left(tp_{i_{IS}}\times tp_{i_{CR}}\right)}{C^{Edge}}$$

(24) $$C4:\sum_{i=1}^{n}\frac{tp_{i_{IS}}}{R_{t}^{R2C}}<\frac{\gamma \times \sum_{i=1}^{n}\left(tp_{i_{IS}}\times tp_{i_{CR}}\right)}{C^{Edge}}$$

## 6. Proposed Offloading and Resource Allocation Model

**Figure 4:**
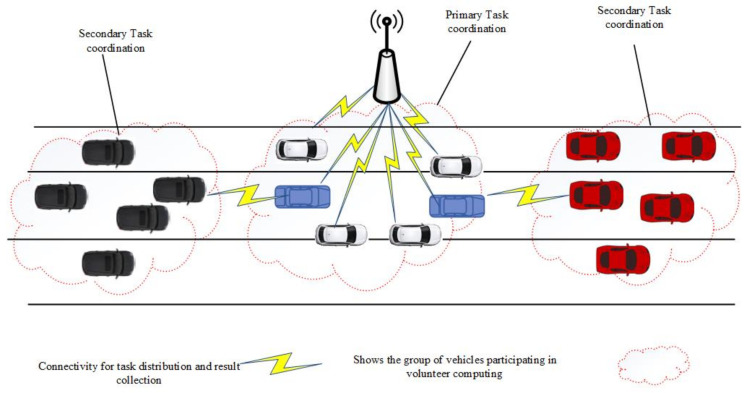
Hybrid VCBV scenario.

In this section, hybrid VCBV is proposed which is used for resource allocation during task execution. We consider a congested road as shown in Figure 4. The solution to the above problem encompasses the strategy of multi-hop task coordination to fully utilize the surplus resources of vehicles beyond the range of an RSU. A decomposition technique is used to fragment the aforementioned problem for solution and optimization. To maximize the system utility the problem is divided into boundary relay vehicles determination (BRVD), hybrid VCBV task coordination (HVTC), and secondary task coordination (STC). We design an algorithm for resource utilization using hybrid VCBV and without using any edge or cloud server.

### 6.1. Boundary Relay Vehicles Determination Algorithm

To achieve the aim of resource utilization in VCBV multi-hop access to volunteers is used. In task coordination, boundary relay vehicles are determined after the identifying the willingness of volunteer vehicles in the communication range of the RSU. These boundary relay vehicles are used to approach the volunteer vehicles which are out-of-the-range of the RSU. The reason to choose the boundary relay vehicles for secondary task coordination is to enhance the region for task coordination. On a congested road, vehicles on both sides of an RSU can play the role of boundary relay vehicles. Each side of the RSU will have exactly one boundary node which will play the role of secondary task coordinator.

Algorithm I is used to determine the boundary relay nodes from a set of volunteers, V. It first computes the distance between the RSU and all the volunteers during the beaconing process. Vehicles with maximum distance but under the communication range of the RSU are boundary relay vehicles for primary task coordination on both sides of the RSU.
**Algorithm I:** Proposed BRVD algorithm for Hybrid VCBV
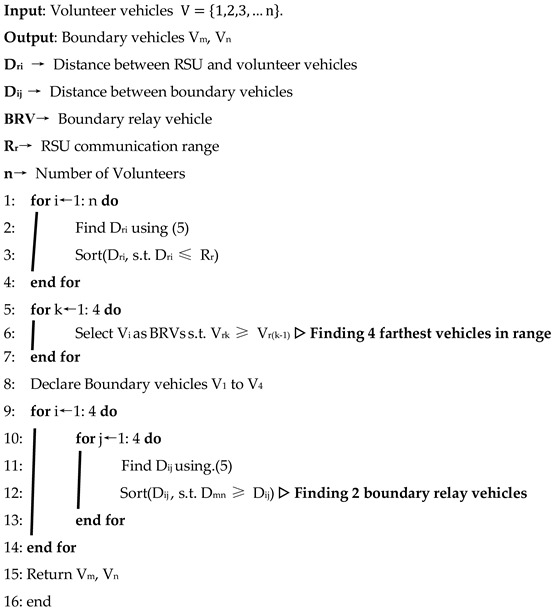



### 6.2. Hybrid Based VCBV Task Coordination Algorithm

As mentioned before, to execute the jobs and utilize the surplus resources of vehicles stuck in traffic congestion, we use hybrid VCBV task coordination. This type of coordination leverages the use of infrastructure as well as ad-hoc coordination simultaneously. To enhance resource utilization and optimize the system utility, hybrid task coordination opts for primary and secondary task coordination. Based on the problem analysis and constraints, the HVTC algorithm is used to maximize the system utility.

We decouple the optimization problem into resource allocation, primary task coordination, determination of boundary relay vehicles, and carrying out the secondary task execution. This algorithm obtains the willingness of n+2 vehicles from which n vehicles are volunteers and two vehicles are boundary relay nodes. It picks three jobs for simultaneous task coordination. The first job is executed using primary task coordination with the resources available in the communication range of the job coordinator. The second and third jobs are offloaded to boundary relay vehicles for secondary task coordination which are allocated to volunteers, not in the range of primary task coordinator. The RSU is responsible for the collection and aggregation of results from primary and secondary task coordination nodes.
**Algorithm II:** Proposed HBVTC algorithm for Hybrid VCBV
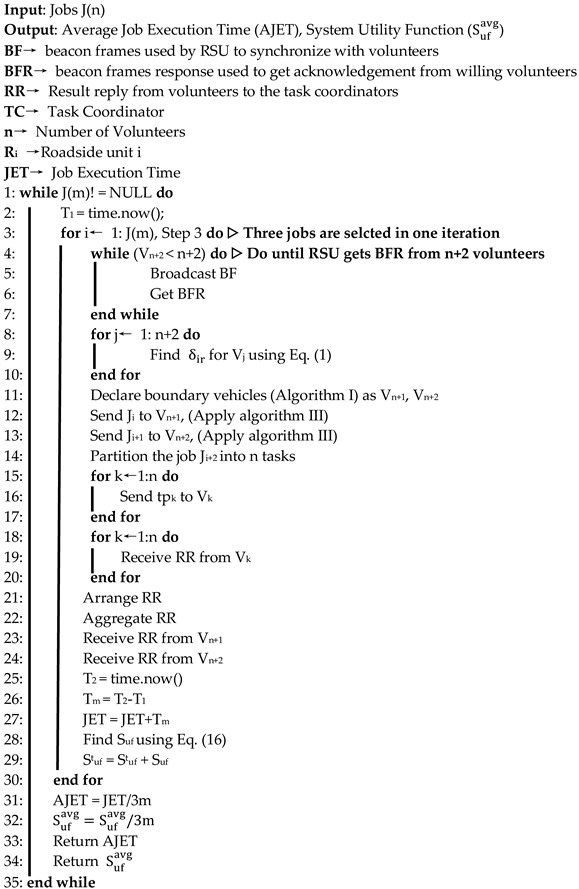



### 6.3. Secondary Task Coordination

This type of coordination is executed in two modes, depending upon the availability of sufficient volunteers. In the first case, it obtains the willingness of n volunteer vehicles, where the boundary relay vehicle acts as a secondary task coordinator. In the second case, on failing to get willingness from sufficient volunteers, the boundary relay vehicle offloads the job to another vehicle willing to be a coordination node. The STC algorithm below shows the whole process of task coordination.
**Algorithm III:** Proposed STC algorithm for Hybrid VCBV
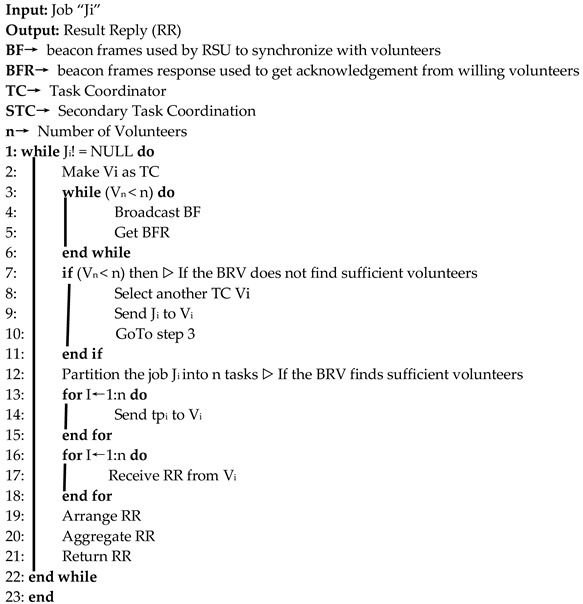



## 7. Performance Evaluations

In this section simulation experiments, conducted in NS3 and Python, are described. The proposed HVTC is evaluated and a performance comparison is made with the following schemes, from which only RVC uses volunteer computing for the execution of jobs.

- *The Entire Local Computing (ELC) scheme*, where all the jobs are executed on the vehicles locally. We take ELC as a benchmark for the decision to offload. Any offloading job expected to have makespan more than ELC will be rejected for the offloading procedure.
- *The Entire Cloud Computing (ECC) scheme*, where all the jobs are offloaded to cloud servers for execution. ECC is modelled using the eDors algorithm [19] which optimizes the consumed energy and latency using dynamic offloading and resource scheduling at the cloud.
- *The Entire Edge Computing (EEC) scheme*, where all the jobs are executed at edge servers. In VEC, these edge servers are placed at RSU and named as VEC servers. We use JSCO [31], a low complexity algorithm to model EEC.
- *The RSU-based VCBV Computing (RVC)* using the single-hop scheme, where all the jobs are executed using volunteers in the communication range of the RSU. This scheme uses infrastructure based VCBV where all the volunteer vehicles are lying in the communication range (one hop) of the RSU.

### 7.1. Simulation Setup

In the simulations, an RSU is placed near a 1000m straight road congested with vehicles as shown in Figure 3. The VEC server and cloud server have computational capabilities of $2\times 10^{10}$ and $1.5\times 10^{11}$ CPU cycles per second, respectively; the vehicles have the computational capability of $1\times 10^{9}$ CPU cycles per second [46]. Backhaul link capacity for cloud ($R_{t}^{R2C}$) is $10^{7}$ bits/s whereas output data size ($D_{o}$) is 200 Kb. α, β, and γ are constants and depend on the availability of RSU to cloud communication bandwidth, cloud, and edge computation capabilities, respectively.

In hybrid VCBV, an RSU obtains the willingness of volunteer vehicles by sending the beacon frames (BFs) and receiving the beacon frame responses (BFRs). A BF contains information regarding the task to be offloaded. From the BFs, the volunteer vehicles acquire the information of required resources and send a BFR to the RSU indicating the availability and willingness of volunteer. After obtaining the willingness of sufficient volunteers, the RSU sends the task data (input data) for executing the computational procedure. After the execution of the assigned task, the results are sent back to the job coordinator.

For RVC and HVTC, we consider one RSU located alongside a two-lane unidirectional road in an urban environment. In our simulation, we consider *n* = 20 vehicles, which may increase in real situations depending upon the willingness of volunteers. We assume these vehicles are in congestion and resource utilization may be accomplished to a higher level depending upon the availability and willingness of volunteers. RVC considers 20 vehicles for coordination and after getting the willingness of these volunteer vehicles, its task coordination is performed. Whereas HVTC considers 22 vehicles for primary coordination and out of these volunteer vehicles, 2 vehicles are considered as boundary relay vehicles which then take part in secondary task coordination. We use NS-3.27 to find the communication costs for initialization, task offloading, and return of results. These results are used for numerical analysis.

Table 2 shows the parameter settings for experimentation.

### 7.2. Performance Comparisons

In this section, we evaluate the performance of ELC, ECC, EEC, RVC, and HTVC in terms of average execution time and system utility parameters for three different scenarios. In the first scenario, the aforementioned parameters are compared to a different number of tasks. In the second scenario, these parameters are analyzed for a fixed number of tasks but different task sizes. In the third scenario, the analysis is conducted for varied computational requirements, for tasks whereas the input size and number of tasks are kept constant.

#### 7.2.1. Different Number of Tasks

**Figure 5:**
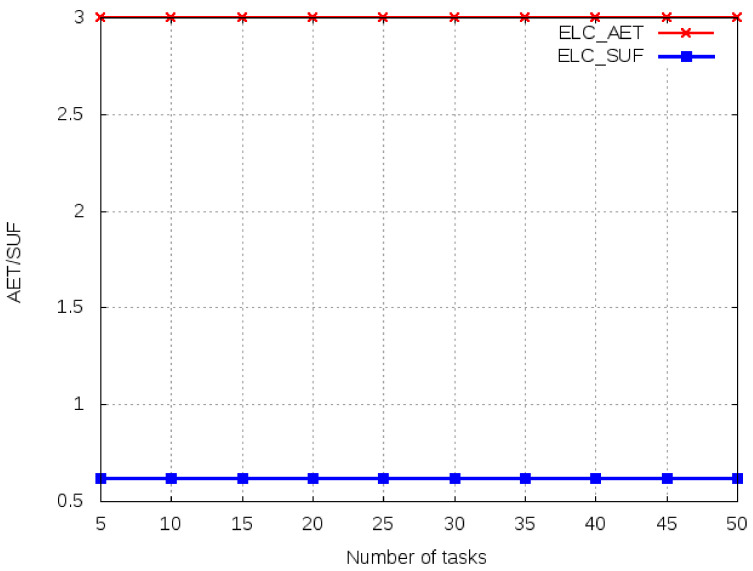
Average execution time and system utility for ELC.

In this scenario, the size of the task is fixed at 1000 Kbits and the number of tasks varies from 10 to 50. We first, compute the average execution time and system utility function for ELC. The performance of ELC is taken as a benchmark for all other computing algorithms. In Figure 5, we observe the benchmark values for average execution time and system utility. Any task with a higher average execution time than ELC will be rejected for offloading for any of the computing algorithms. Task execution time increases with an increase in the number of tasks, but average execution time remains constant for several tasks due to the fixed computation requirements and similar OBU types.

**Figure 6:**
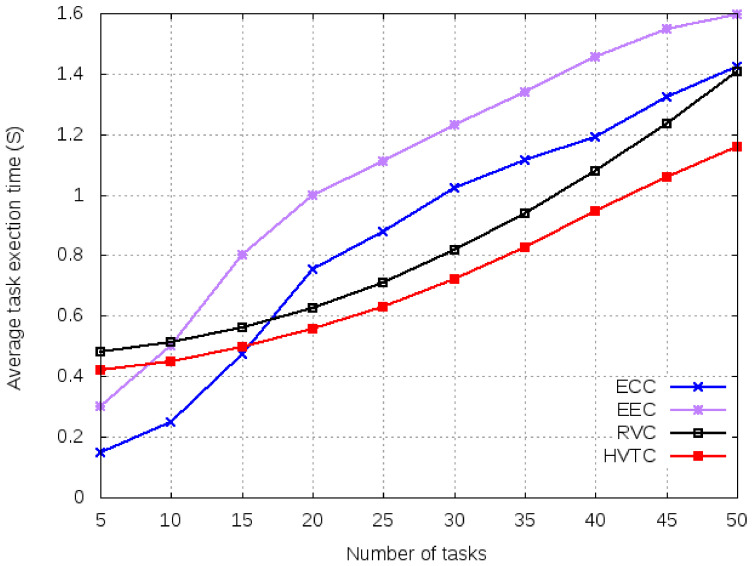
Average execution time for a varied number of tasks.

It is observed in Figure 6 that the average execution time for a small number of tasks is lower when using cloud or edge mechanisms over VCBV algorithms. The reason for this good performance is due to the higher computation resources of cloud and edge computing than the OBUs within vehicles. As the number of tasks increases, the performance of ECC and EEC decreases due to communication and computation constraints. Both RVC and HVTC use volunteer computing for resource allocation but differ in the number of hops. HVTC shows better performance than RVC. The reason behind this is better resource allocation in HVTC, due to multi-hop communication. Even for a smaller number of tasks, HVTC uses three times more resources. It uses the same number of volunteers as is used in RVC, only in primary coordination. Using a multi-hop resource allocation increases the number of volunteers resulting in lower computation time. This technique optimizes the makespan but occupies more communication resources during the offloading process.

**Figure 7:**
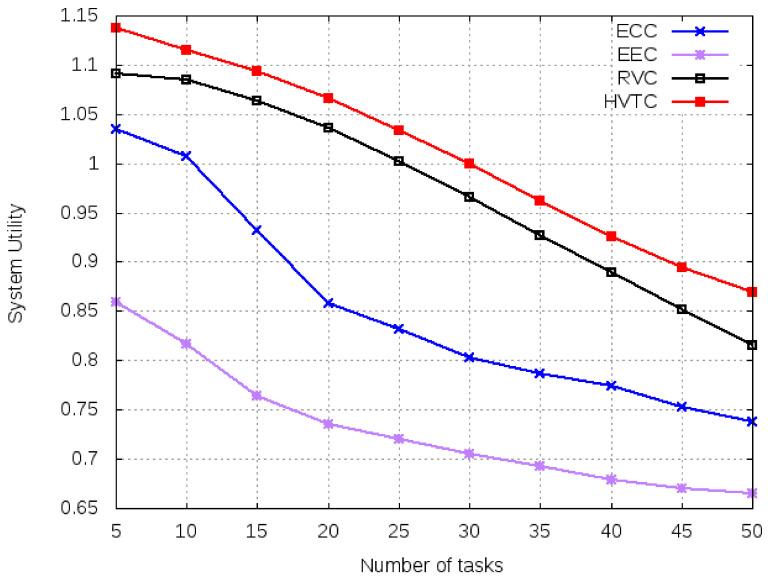
System utility for a varied number of tasks.

Figure 7 shows the simulation results of the system utility function for computing algorithms for a varying number of tasks. The system utility of any computing algorithm depends on the makespan and cost paid to third party vendors such as cloud and edge provision. The lower the makespan and monetary cost, the higher the system utility of the algorithm. The reason to use system utility for comparison is to highlight the importance of free of cost computing services. RVC and HTVC have better performance and higher system utilities comparing to ECC and EEC because RVC and HTVC use volunteer computing and charge no monetary cost.

#### 7.2.2. Varied Task Size

For near-optimal solution, effective computation offloading relies on the makespan which comprises communication and computation delays. Computation cost can be optimized by using more resources from the cloud, edge, or volunteer resources. Similarly, communication cost depends on the size of input and output data. We have performed experiments to determine the effect of varied task size on communication and computation costs. First, we perform local computation and analyze the effect of varied data size on average execution time and system utility.

**Figure 8:**
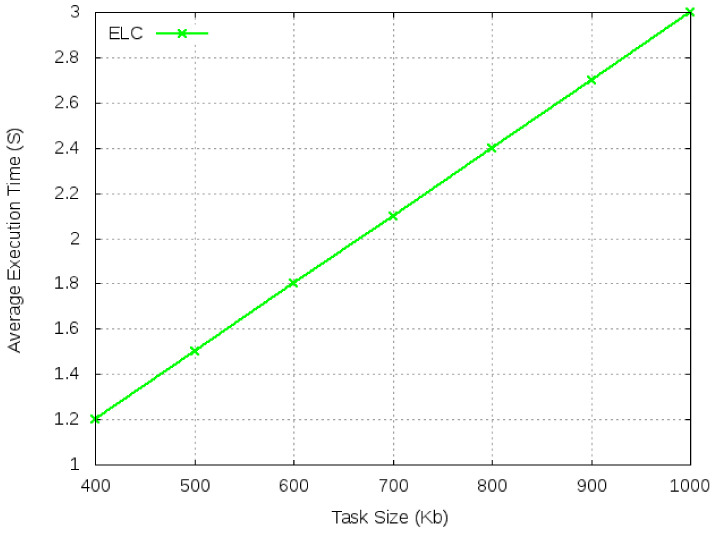
Average execution time for ELC with varied task size.

**Figure 9:**
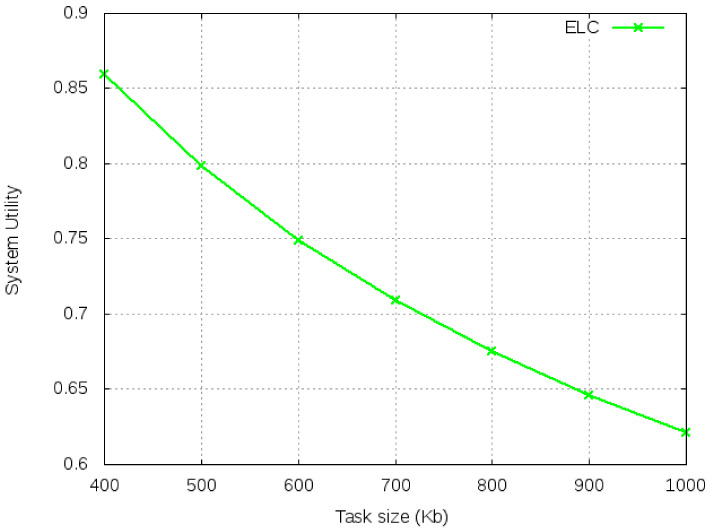
System utility for ELC with varied task size.

Figure 8 and Figure 9 show the benchmark performance for average execution time and system utility for various sizes of task. We observe that with the increase in input data size, the average execution time increases while system utility decreases.

**Figure 10:**
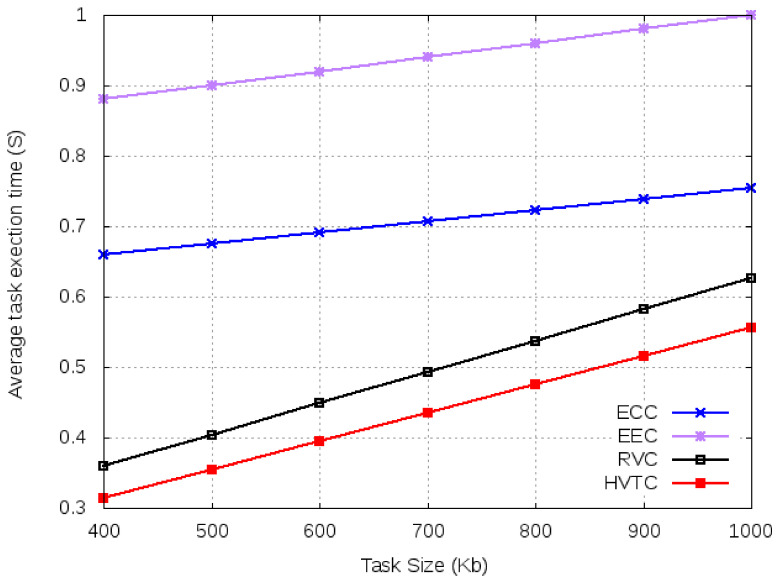
Average execution time for varied task size.

**Figure 11:**
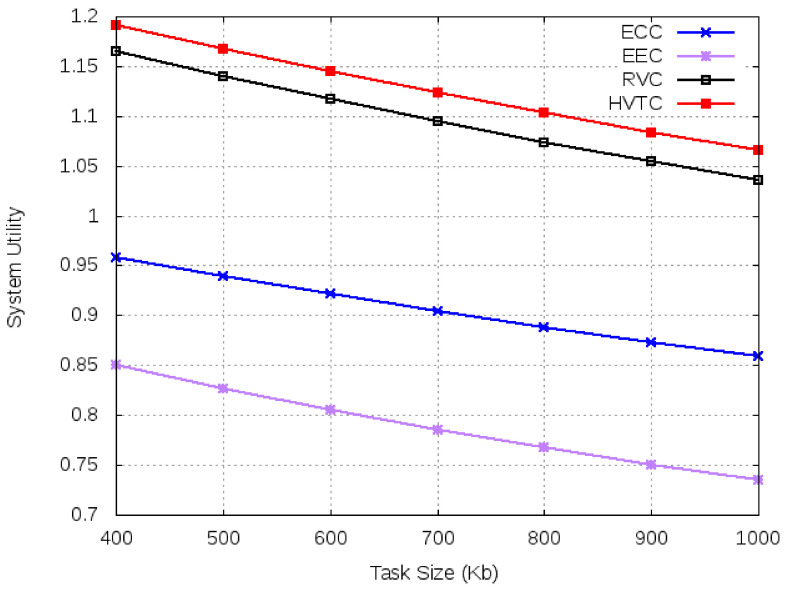
System utility for varied task size.

For simulations, we fix the number of tasks at 20 and vary the task input data size from 400 Kb to 1000 Kb. From Figure 10, it can be observed that EEC and ECC have higher average execution time compared to volunteer computing-based algorithms. An increase in the number of tasks affects the performance of edge and cloud due to increase communication and computation requirements. However, a task with a smaller input size has a lower execution time. According to Figure 11, ECC and EEC have smaller system utility compared to volunteer computing-based algorithms.

#### 7.2.3. Varied Computational Requirements

Task offloading in vehicular networks is usually performed for two reasons. The first, is when the processing requirements of a task is more than the computational capacity of a vehicle. Secondly, when there is some requirement of a deadline to meet which is not possible with ELC due to the higher makespan. The decision to offload a task or not usually depends on the ratio of computing to communication costs. The third factor on which makespan incurred for task offloading depends is the task computational requirements. In this scenario, the size of the task and number of tasks are fixed to 1000 Kbits and 20 respectively. We vary task computational requirements from 150 to 1500 CPU cycles per bit. These computational requirements are investigated for the different type of workloads from data to video processing tasks [49].

**Figure 12:**
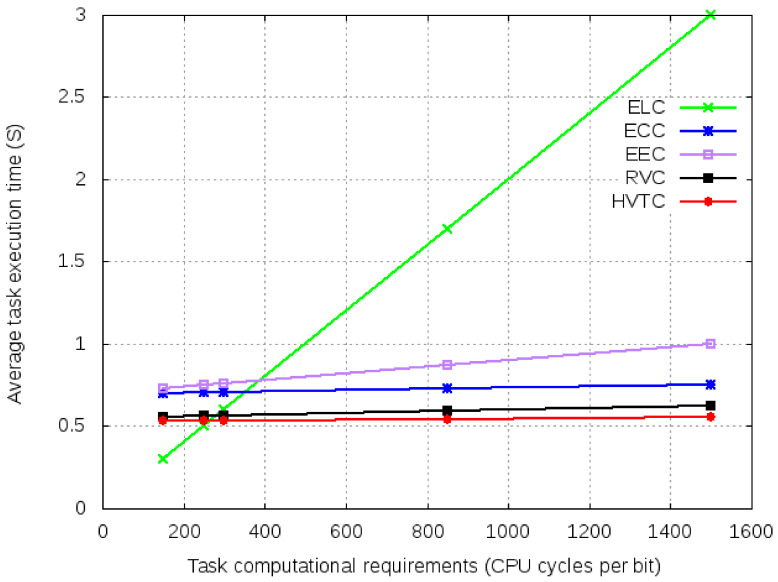
Average execution time for varied task computational requirements.

From Figure 12, it is observed that the tasks with computation requirements less than 300 CPU cycles per bit have better average execution time than offloading to other devices. Here communication cost incurred due to offloading is greater than the time required for ELC. RVC and HVTC show better performance than the other offloading techniques. Since HVTC incurs additional offloading overheads, it has almost the same performance as RVC for tasks having fewer computational requirements. Similarly, EEC has better performance due to the only dependency being on computational requirements.

**Figure 13:**
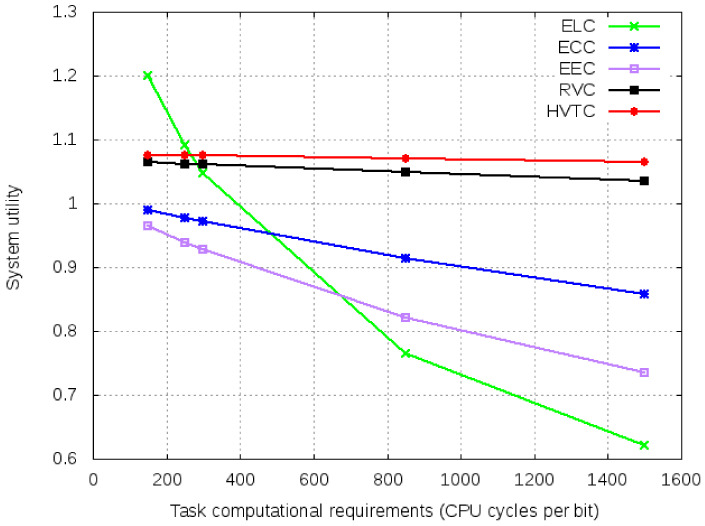
System utility for varied task computational requirements.

Figure 13 shows the system utility values for varied task computational requirements. ELC shows better system utility because it does not involve offloading and monetary costs. Even with lower computational capabilities, it shows better performance for tasks with low computational requirements. HVTC has the highest system utility except for the task with computational requirements of less than 300 CPU cycles per bit. Like ELC it does not involve the monetary cost, but it has a communication cost for offloading.

## 8. Conclusions

In this article, we have proposed a hybrid volunteer computing-based model in vehicular networks to minimize latency and maximize system utility. We achieve this by utilizing the surplus resources in vehicular networks. In particular, the surplus resources of vehicles in congestion are considered for efficient utilization. The volunteer model not only optimizes the latency but reduces the monetary costs required for task offloading to third party vendors. We analyze the task coordination model in a single and multi-hop fashion by using boundary relay nodes which minimize the need for additional infrastructures. Extensive simulations are performed to validate the performance of the hybrid coordination model which show that hybrid VCBV is not only better in latency but shows a higher system utility over existing schemes. It saves on the financial costs used to employ task offloading services, utilizes surplus resources, and achieves a lower makespan given sufficient availability and willingness of volunteers. The VCBV model supplements edge and cloud technologies and minimizes third-party reliance. Our proposed model considers the resource utilization of vehicles stuck in congestion in an urban environment. In future, we will consider the resource utilization of vehicles moving on highways using game theory.

## Figures and Tables

**Table 1:** List of notations.

Notation	Description
V, n	Set/number of volunteer vehicles
$\delta_{ir}$	Distance of node i from the boundary
$D_{ir}$	Distance between vehicle i and RSU
$R_{t}^{V2V},\;R_{t}^{V2R}$	Data transmission rate for the wireless channel between V2V and V2R
$R_{r}$	Communication range of an RSU
tpi	A tuple representing the task allocated to vehicle i
$tp_{i_{ID}}$	Identity of the task sent to vehicle i
$D_{o}$	Output data size
$tp_{i_{IS}}$	Input size of the task allocated to vehicle i
$tp_{i_{CR}}$	Required computational resources for computing task $t_{i}$
$C_{i}^{Veh}$	The computational capability of the volunteer vehicle
$C^{CC}$	The computational capability of the cloud
$C^{Edge}$	The computational capability of the edge
$T_{tp_{i}}^{comp}$	Time taken by a task to complete execution on an OBU
$T_{j}$	Makespan for job *j*
$T_{avg}^{exec}$	Average execution time for all m jobs
$T_{i}$	The total time taken for a task from transmission time to completion of task
$J_{i}$	Number of jobs for vehicle i
$W_{i}$	The number of tasks a vehicle i has for execution
$O_{1},\;O_{2},\;O_{3}$	Objective functions
α, β, γ	Constants used for differentiation of available computation capabilities
LET	Link expiration time
$V_{i}$	Vehicle i
$S_{uf}$	System utility function

**Table 2:** Parameter Settings.

Notation	Description	Values
BF	Hello packet size	20 B
TA	Data packet size	1000 B
$\sum_{i=1}^{n}tp_{i}{}_{IS}$	Input data size [47]	[400,1000] Kb
$D_{o}$	Output data size [47]	[50,200] Kb
$D_{o_{i}}$	Output data size for $tp_{i_{IS}}$	[2,10] Kb
$C_{i}^{Veh}$	Vehicle computation capacity [47]	$\left[1\times 10^{9},\;2\times 10^{9}\right]$ CPU cycles/s
$C^{Edge}$	Edge computation capacity [46]	$\left[2\times 10^{10},\;8\times 10^{10}\right]$ CPU cycles/s
$C^{CC}$	Cloud computation capacity [46]	$\left[1.5\times 10^{11},\;6\times 10^{11}\right]$ CPU cycles/s
$R_{t}^{R2C}$	Backhaul link capacity [46]	[$5\times 10^{6}$, $50\times 10^{6}$] bits/s
$R_{v}$	Communication Range of vehicles [48]	150 m
$R_{r}$	Communication Range of RSU [48]	200 m
$P_{c}^{\mathrm{Cloud}}$	Computation resource cost at cloud [30]	$0.015/GHz
$P_{c}^{\mathrm{Edge}}$	Computation resource cost at edge [30]	$0.03/GHz
$tp_{i_{CR}}$	Task computational requirements [47]	1500 CPU cycles per bit

## Data Availability

Not applicable.
